# PGC‐1α, a potential therapeutic target against kidney aging

**DOI:** 10.1111/acel.12994

**Published:** 2019-07-16

**Authors:** Gayoung Lee, Md Jamal Uddin, Yoojeong Kim, Minji Ko, Inyoung Yu, Hunjoo Ha

**Affiliations:** ^1^ Graduate School of Pharmaceutical Sciences Ewha Womans University Seoul Korea; ^2^ College of Pharmacy Ewha Womans University Seoul Korea

**Keywords:** kidney aging, lipid metabolism, mitochondria, peroxisome, PGC‐1α

## Abstract

Aging is defined as changes in an organism over time. The proportion of the aged population is markedly increasing worldwide. The kidney, as an essential organ with a high energy requirement, is one of the most susceptible organs to aging. It is involved in glucose metabolism via gluconeogenesis, glucose filtration and reabsorption, and glucose utilization. Proximal tubular epithelial cells (PTECs) depend on lipid metabolism to meet the high demand for ATP. Recent studies have shown that aging‐related kidney dysfunction is highly associated with metabolic changes in the kidney. Peroxisome proliferator‐activated receptor gamma coactivator‐1 alpha (PGC‐1α), a transcriptional coactivator, plays a major role in the regulation of mitochondrial biogenesis, peroxisomal biogenesis, and glucose and lipid metabolism. PGC‐1α is abundant in tissues, including kidney PTECs, which demand high energy. Many in vitro and in vivo studies have demonstrated that the activation of PGC‐1α by genetic or pharmacological intervention prevents telomere shortening and aging‐related changes in the skeletal muscle, heart, and brain. The activation of PGC‐1α can also prevent kidney dysfunction in various kidney diseases. Therefore, a better understanding of the effect of PGC‐1α activation in various organs on aging and kidney diseases may unveil a potential therapeutic strategy against kidney aging.

## 1 INTRODUCTION

The geriatric population is explosively increasing worldwide (Commission, 2015). Approximately 617 million people (8.5% of people worldwide) are 65 years or older. This number is estimated to increase to 1.6 billion by 2050 (Kowal, Goodkind, & He, 2016). With an increasing population of the elderly, healthy aging has emerged as a crucial issue. Aging is a progressive disruption of the homeostasis of physiological systems with age. It results in structural destruction, organ dysfunction, and increased susceptibility to injuries and diseases. The kidney is one of the most susceptible organs to aging (Wang, Bonventre, & Parrish, 2014). Aging‐associated complications can lead to kidney dysfunction, including a decreased glomerular filtration rate (GFR), tubular dysfunction, and glomerulosclerosis. Furthermore, kidney aging has important implications for aging‐associated comorbidities, especially cardiovascular diseases. While the molecular mechanism underlying kidney aging remains unclear, chronic kidney disease (CKD) shares many phenotypic similarities with aging, including cellular senescence, fibrosis, vascular rarefaction, loss of glomeruli, and tubular dysfunction (Kooman, Kotanko, Schols, Shiels, & Stenvinkel, 2014). The pathogenic mechanisms involved in CKD may thus provide insight into the molecular pathways leading to kidney aging. They might also provide potential targets against kidney aging. Recent efforts to overcome aging have shifted from the identification of risk factors to the determination of endogenous protective factors that might neutralize the adverse effects of aging. Among the various endogenous protective factors reported (Jeong & King, 2011), AMP‐activated protein kinase (AMPK) (Casalena, Daehn, & Bottinger, 2012; Kume, Thomas, & Koya, 2012; Sharma, 2015), fibroblast growth factor 21 (FGF21) (Salminen, Kaasniranta & Kauppinen, 2017), insulin (Artunc et al., 2016), and vascular endothelial growth factor (VEGF) (Schrijvers, Flyvbjerg, & De Vriese, 2004) have been extensively reviewed and are briefly summarized in Table 1. Pyruvate kinase isozyme type M2 (PKM2) has recently been suggested as an endogenous protective factor against diabetes‐induced kidney injury (Qi et al., 2017). This review aimed to discuss current data on endogenous PGC‐1α as a potential therapeutic target against kidney aging.

**Table 1:** Effects of various endogenous protective factors on the kidney

Endogenous protective factors	Protective effect/mechanism	References
AMPK	Glomerulus—increases autophagy and mitochondrial biogenesis; reduces apoptosis; reduces oxidative stress Tubule—increases autophagy, fatty acid oxidation, and mitochondrial biogenesis; reduces oxidative stress	Decleves, Mathew, Cunard, and Sharma (2011), Decleves et al. (2014), Dugan et al. (2013), Fang et al. (2013), Jin, Liu, Ma, Xiao, and Chen (2017), Sharma et al. (2008), Sohn et al. (2017)
FGF21	Glomerulus—maintains differentiated podocytes; reduces oxidative stress Tubule—reduces apoptosis and oxidative stress; increases autophagy	Davidson, Dono, and Zeller (2001), Kim, Lim, et al. (2013), Li, Liu, Tang, Cai, and Dong (2018), Minami et al. (2017), Zhang, Shao, et al. (2013), Zhang, Zhou, et al. (2013)
Insulin	Glomerulus—maintains the integrity of the glomerular filtration barrier through cytoskeletal reorganization; reduces mesangial cell apoptosis Tubule—inhibits gluconeogenesis in the proximal tubules; increases Na reabsorption in the distal tubules	Hiromura et al. (2002), Tiwari et al. (2008, 2013), Welsh et al. (2010)
PKM‐2	Glomerulus—increases glucose metabolic flux and mitochondria metabolism; inhibits the production of toxic glucose metabolites in podocytes	Qi et al. (2017)
VEGF	Glomerulus—reduces apoptosis; maintains podocyte foot processes and endothelial cells fenestration; increases endothelial cells proliferation; preserves the glomerular capillary endothelium Tubule—reduces apoptosis; preserves the peritubular capillary endothelium	Harvey, Engel, and Chade (2016), Kanellis Fraser Katerelos & Power (2000), Kang, Hughes, Mazzali, Schreiner, and Johnson (2001), Kim et al. (2000), Sison et al. (2010)

## 2 FEATURES OF KIDNEY AGING

**Figure 1:**
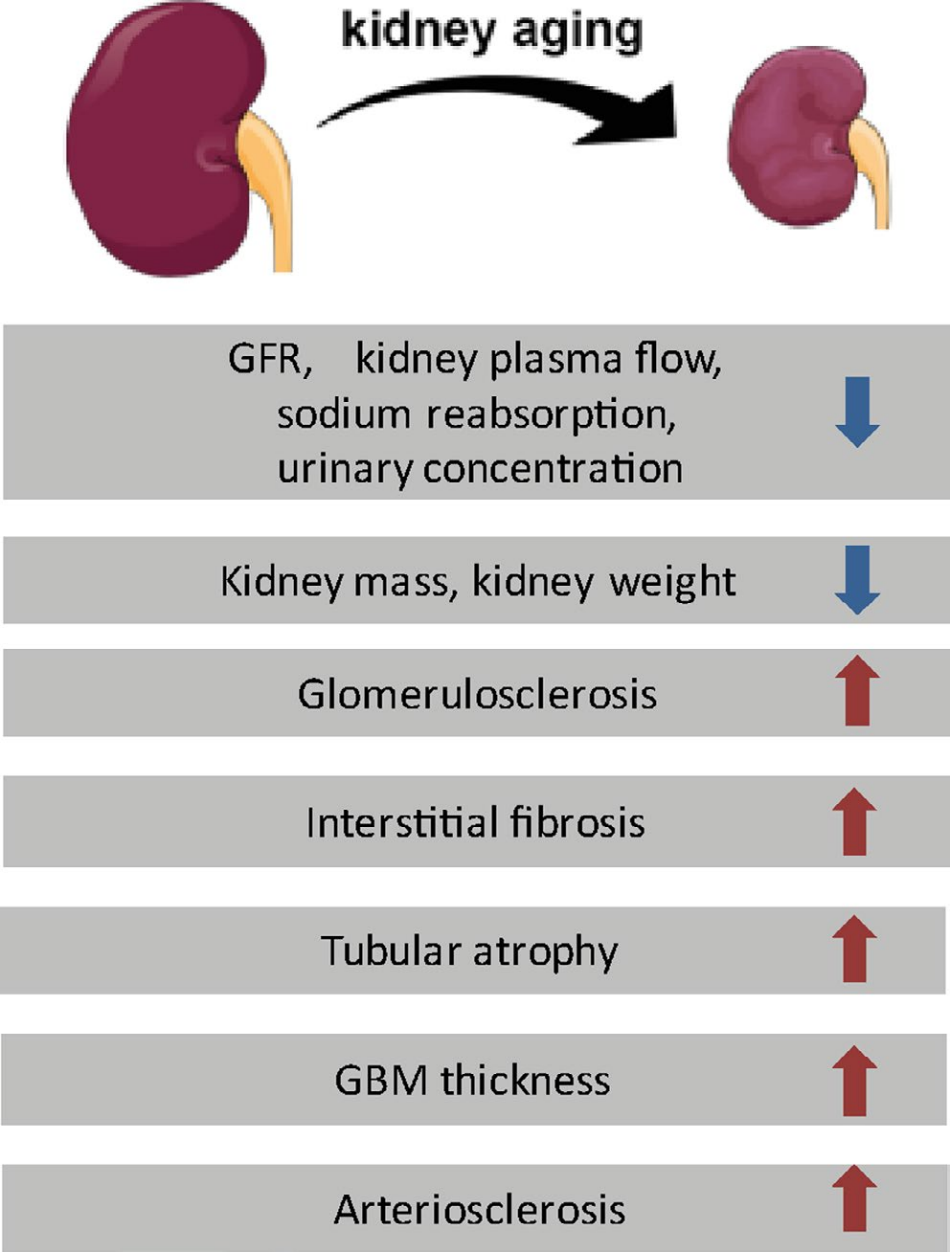
Age‐associated changes in the kidney. Altered macroscopic and microscopic changes decrease kidney function during the aging process. GBM, glomerular basement membrane

This section briefly summarizes the features of kidney aging (Figure 1), a complex process affected by various factors including chronic inflammation, oxidative stress, genetics, and accompanying chronic diseases such as diabetes and hypertension (Kaplan, Pasternack, Shah, & Gallo, 1975). In the seventh decade of life, the kidney mass is 20%–30% less than that in the fourth decade. Such an age‐related reduction is more pronounced in the cortex than in the medulla (Gourtsoyiannis, Prassopoulos, Cavouras, & Pantelidis, 1990; Hoy et al., 2003). Pathological fibrosis, a representative hallmark of aging, is also observed in kidney aging. Disruptions and changes in normal kidney structure may be accelerated by aging‐induced profibrotic signals such as transforming growth factor‐β (TGF‐β) (McLachlan, 1978; Yang et al., 2017). Progressive tubular dysfunction is accompanied by decreased sodium reabsorption and potassium excretion with reduced urine concentrating capacity. Because podocytes have very limited regenerative potential, the excessive atrophy of podocytes contributes to glomerular hyperfiltration (Wiggins et al., 2005). Alterations in kidney vasculature, such as intimal and medial hypertrophy and arteriosclerosis in afferent arterioles, have been observed in kidney aging (Michelis, 1990). A massive loss of functional glomeruli with age also results in the irregularity and tortuosity of afferent arterioles and direct connections between afferent and efferent vessels, leading to blood flow bypassing the glomeruli (Kaplan et al., 1975). The aging kidney also exhibits a gradual reduction in kidney plasma flow, mainly in the kidney cortex. Kidney vascular responses to endogenous vasodilators such as nitric oxide, atrial natriuretic peptide, and amino acids are decreased, whereas the sympathetic tone and response to angiotensin II are increased (Wiggins et al., 2005). As a result, vasoconstriction increases with age (Takazakura et al., 1972).

## 3 METABOLISM IN KIDNEY AGING AND CKD

The kidney is not generally considered a major metabolic organ, although it contributes to glucose metabolism via gluconeogenesis, glucose filtration and reabsorption, and glucose utilization (Alsahli & Gerich, 2017). The kidney accounts for approximately 10% of all glucose utilized by the body under normal conditions. Under normal conditions, 180 g/day of glucose is filtered by the kidney glomerulus and reabsorbed in the proximal tubule (Gerich, 2010). Gluconeogenesis in the kidney contributes up to 25% of systemic glucose levels under normal conditions (Mather & Pollock, 2011). Patients with type 2 diabetes mellitus (T2DM) exhibit increased glucose production (up to 300%) and glucose uptake by the kidney (Alsahli & Gerich, 2017).

Insulin plays an important role in glucose homeostasis. Insulin receptor (IR) is expressed throughout the kidney, suggesting an important role of insulin in the kidney. Under insulin resistance, an impaired insulin cascade is observed not only in classical insulin target tissues (liver, skeletal muscle, and white adipose tissue) but also in the kidney (Horita et al., 2016). In the kidney of T2DM patients, the IRS1‐dependent inhibition of gluconeogenesis is impaired in proximal tubules (Horita et al., 2016), while IRS2‐dependent signaling is preserved in proximal and distal tubules, leading to hypertension through increased sodium reabsorption (Artunc et al., 2016). Proximal tubule‐specific insulin‐resistant‐knockout mice show hyperglycemia through increased gluconeogenesis (Tiwari et al., 2013), whereas podocyte‐specific insulin‐resistant‐knockout mice show losses of podocyte foot processes and cytoskeletal architecture and develop significant albuminuria under normoglycemic conditions (Welsh et al., 2010). An impairment in IRS1 signaling induces podocyte dysfunction and deteriorates the podocyte structure, which may induce diabetic kidney injury (Welsh et al., 2010). In addition, insulin confers protection from apoptotic stimuli by stimulating the PI3K‐Akt pathway in mesangial cells (Hiromura, Monkawa, Petermann, Durvasula, & Shankland, 2002).

Kidney proximal tubules have high levels of baseline energy consumption (Kang et al., 2015; Meyer, Nadkarni, Stumvoll, & Gerich, 1997). Fatty acid oxidation (FAO) is the preferred energy source in proximal tubules because fatty acid (FA) generates more ATP than glucose at an equal molar concentration during oxidation. In fact, the kidney cortex has low glucose‐phosphorylating capacity but high levels of oxidative enzymes, supporting that the kidney cortex uses free fatty acids (FFAs) and not glucose as the main source of energy (Gerich, 2010). The kidney medulla uses glucose anaerobically for its energy requirement due to its low levels of oxidative enzymes.

Lipoprotein lipase and CD36 are two important molecules for FA uptake. Cytosolic FAs can be supplied either by in situ cytosolic synthesis or by the deacylation of cellular phospholipids through the action of phospholipase A2 (Simon & Hertig, 2015). FAs are then transported from the cytosol to respective organelles (mitochondria and peroxisomes) to be oxidized to provide cells with ATP. The mitochondrial transporter system consists of two components: carnitine palmitoyltransferases (CPT1 and CPT2) and a carnitine‐acylcarnitine translocase. The peroxisome transporter system requires three ATP‐binding cassette transporter D subfamily proteins: ABCD1, ABCD2, and ABCD3 (Wanders, 2013). Human kidney samples with diabetic nephropathy show lipid accumulation in the glomeruli and tubulointerstitium along with the upregulation of CD36 (Herman‐Edelstein, Scherzer, Tobra, Levi & Gafter, 2014; Hua et al., 2015).

A defective FAO pathway induces lipid accumulation, resulting in lipotoxicity that contributes to the development of CKD in humans and rodents (Chung et al., 2018; Hager, Narla, & Tannock, 2017; Han et al., 2016; Kang et al., 2015; Nitta, 2012). These results suggest that the proper adaptation of FAO is an important strategy against kidney aging. A recent lipidomic analysis revealed significant age‐related differences in lipid metabolites of the kidney (Braun et al., 2016). In addition, large cohort studies have demonstrated that distinct metabolomic signatures, including lipid metabolism, are associated with longevity in humans (Cheng et al., 2015).

PGC‐1α and peroxisome proliferator‐activated receptor α (PPARα) are important in glucose metabolism and act as master regulators of lipid metabolism by regulating mitochondrial and peroxisomal FAO‐related genes (Chung et al., 2018). While the interaction between these two proteins has been well established, PGC‐1α can also coactivate PPARδ, which induces FAO (Kleiner et al., 2009). The levels of PGC‐1α, PPARα, and FAO‐associated enzymes are reduced in aged kidneys with significantly increased lipid accumulation (Chung et al., 2018; Lim et al., 2012). PGC‐1α‐related FAO genes are reduced in CKD. The overexpression of PGC‐1α ameliorates Notch‐induced kidney fibrosis, a phenotype in aging.

## 4 PGC‐1α: A KEY PLAYER IN METABOLISM

**Figure 2:**
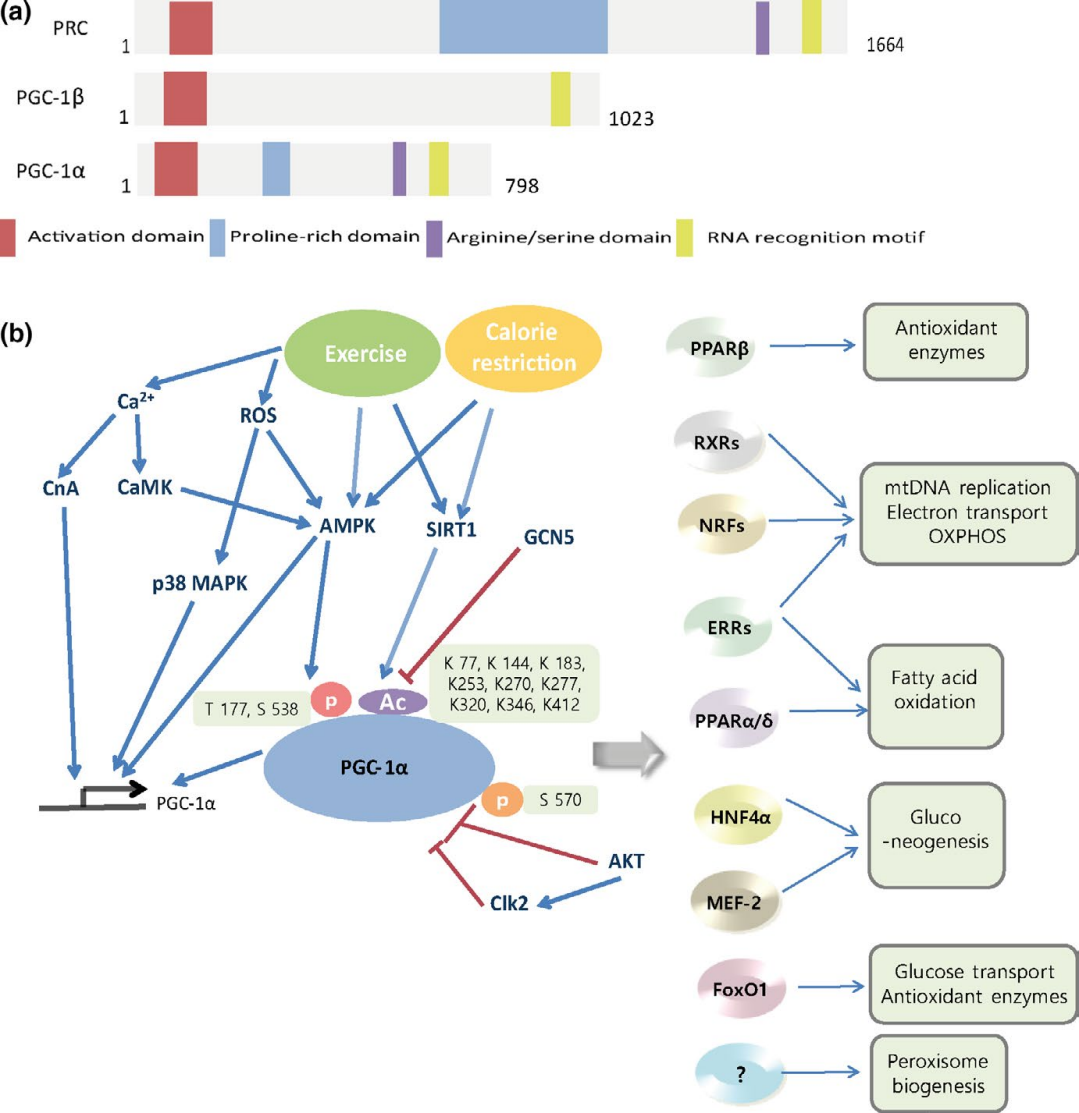
(a) Domain structure of PGC‐1 coactivators. (b) Upstream regulator and target of PGC‐1α. Ca^2+^, ROS, SIRTs, AMPK, and Akt can regulate the expression and/or activity of PGC‐1α. PGC‐1α then coactivates transcription factors such as NRFs, ERRs, and PPARs, which regulate different aspects of energy metabolism, including mitochondrial biogenesis, peroxisomal biogenesis, fatty acid oxidation, and antioxidant activity. CaMK, Ca2+/calmodulin‐dependent protein kinase; CnA, calcineurin; GCN5, general control of amino acid synthesis 5; ROS, reactive oxygen species; RXR, retinoid receptor

PGC‐1α plays a central role in the regulation of metabolism. It belongs to the PGC‐1 family comprised of PGC‐1α, PGC‐1β, and PGC‐related coactivator (Figure 2a) (Lynch, Tran, & Parikh, 2018). The PGC‐1 family shares sequence homology in the activation domain, the proline‐rich domain, the Arg/Ser‐rich domain, and the RNA‐binding domain of the gene. PGC‐1β lacks the proline‐rich and Arg/Ser‐rich domains (Scarpulla, 2011). Although these proteins show structural and functional similarities, PGC‐1 family members have different tissue distributions (Vega, Huss, & Kelly, 2000). PGC‐1α is expressed abundantly in kidney tubular epithelial cells, whereas PGC‐1β is barely present in the kidney (Liang & Ward, 2006; Lin, Puigserver, Donovan, Tarr, & Spiegelman, 2002; Rasbach & Schnellmann, 2007). Thus, PGC‐1α has been extensively studied in kidney cells and tissues among PGC‐1 family members.

PGC‐1α was first discovered in 1998 as an inducible thermogenic regulator in brown fat and skeletal muscle upon the exposure of mice to cold (4°C) (Puigserver et al., 1998). PGC‐1α expression is increased in response to physical activity, nutritional deficiency, hypoxia, cyclic adenosine monophosphate activation, and oxidant stress (Lynch et al., 2018). PGC‐1α is critical for maintaining energy homeostasis. PGC‐1α regulates cold‐induced thermogenesis, mitochondrial biogenesis, hepatic gluconeogenesis, and FAO (Cheng, Ku, & Lin, 2018; Ventura‐Clapier, Garnier, & Veksler, 2008). PGC‐1α regulates oxidative phosphorylation by targeting genes involved in the subunits of the respiratory chain, including β‐ATP synthase, cytochrome C oxidase (COX) IV, and cytochrome C (Finck & Kelly, 2006). PGC‐1α partners, including PPARs (PPARα and PPARδ), estrogen‐related receptor (ERR), hepatic nuclear factor 4*α* (HNF4*α*), forkhead box protein O1 (FOXO1), nuclear respiratory factor 1 (NRF1), and myocyte enhancer factor 2 (MEF2), have been identified (Fernandez‐Marcos & Auwerx, 2011; Puigserver et al., 2003; Semple et al., 2004) (Figure 2b), indicating that this coactivator can serve as a regulator of multiple pathways involved in cellular energy metabolism.

The role of AMPK, a well‐recognized upstream regulator of PGC‐1α, in kidney disease and aging has been extensively reviewed elsewhere (Casalena et al., 2012; Kume et al., 2012; Sharma, 2015). In brief, AMPK is inhibited in pathological conditions such as inflammation, diabetes, and aging. The activation of AMPK has beneficial effects on these conditions. AMPK can also critically regulate mitochondrial functions linked to multiple pathways involved in aging. AMPK enhances mitochondrial biogenesis not only by inducing the transcription of PGC‐1α but also by activating PGC‐1α by the phosphorylation of threonine‐177 and serine‐538 (Jager, Handschin, St‐Pierre, & Spiegelman, 2007; Jorgensen et al., 2005; Suwa, Nakano, & Kumagai, 2003). Excessive mitochondrial superoxide under hyperglycemic stress is generally acknowledged as the driver of diabetic vascular and kidney injury (Brownlee, 2005). However, this hypothesis was recently challenged by the concept of mitochondrial hormesis (Sharma, 2015). Excessive glucose or nutrients can reduce mitochondrial superoxide production, oxidative phosphorylation, and ATP generation in the target tissues of diabetic complications. A persistent reduction in oxidative phosphorylation in the mitochondria may trigger reactive oxygen species (ROS) generation in nonmitochondrial compartments and the upregulation of proinflammatory and profibrotic cytokines (Coughlan & Sharma, 2016; Dugan et al., 2013). As expected, the activation of AMPK can restore mitochondrial function and superoxide production (Sharma, 2015), underscoring the importance of maintaining physiological mitochondrial superoxide production.

TGF‐β plays a pivotal role in the development of fibrotic tissue, a common hallmark of aging. Interestingly, AMPK activation can downregulate TGF‐β transcriptional activity in various tissues, such as the kidney, liver, and lung. AMPK also inhibits the interaction between the transcription coactivator p300 and SMAD3 in hepatic stellate cells (Casalena et al., 2012). Thus, AMPK might serve as a potential target against kidney aging.

Akt and sirtuin 1 (SIRT1) are major upstream regulators of PGC‐1α. The activation of Akt induces the phosphorylation of PGC‐1α at serine‐570, a reduced form of activated PGC‐1α (Li, Monks, Ge, & Birnbaum, 2007). Recently, it was demonstrated that cdc2‐like kinase (Clk2) protein levels and kinase activity can be induced by the insulin/Akt pathway. Clk2 directly phosphorylates the SR domain on PGC‐1α, resulting in the repression of PGC‐1α expression (Rodgers, Haas, Gygi, & Puigserver, 2010). In addition, 13 conserved arginines of PGC‐1α are sites of inhibitory acetylation by the acetyltransferase GCN5. The deacetylation and reactivation of PGC‐1α are mediated by SIRT1 and are associated with longevity (Canto & Auwerx, 2009; Rodgers, Lerin, Gerhart‐Hines, & Puigserver, 2008). In response to fasting, SIRT1 deacetylates PGC‐1α to control gluconeogenic and glycolytic gene expression in a nicotinamide adenine dinucleotide (NAD)‐dependent pathway (Rodgers et al., 2005). PGC‐1α is a coactivator of the transcription factors NRF‐1 and NRF‐2, which regulate the expression of TFAM, a nuclear‐encoded transcription factor essential for replication, maintenance, and the transcription of mitochondrial DNA. NRF‐1 and NRF‐2 also control the expression of nuclear genes encoding respiratory chain subunits and proteins required for mitochondrial function (Lin, Handschin, & Spiegelman, 2005; Schreiber et al., 2004; Wu et al., 1999). Both AMPK and SIRT1 are major metabolic signaling pathways activated under calorie restriction (CR) (Martin‐Montalvo & de Cabo, 2013), the most effective measure to prevent age‐associated diseases and extend longevity.

Several pathways are involved in PGC‐1α expression in response to exercise: Ca2+/calmodulin‐dependent protein kinase (CaMK), calcineurin (CnA), AMPK, ROS, and NAD. CnA interacts with and activates MEF2, which subsequently induces PGC‐1α transcription (Handschin, Rhee, Lin, Tarr, & Spiegelman, 2003). The Ca2+‐induced CaMK phosphorylation and activation of CREB induce PGC‐1α transcription (Handschin et al., 2003). CaMK is also upstream kinase of AMPK. AMPK is activated by contractile activity in skeletal muscle (Fujii et al., 2000). Activation of the p38 MAPK pathway after exercise stimulates PGC‐1α promoter activity (Akimoto et al., 2005). ROS are also functionally important for exercise‐induced PGC‐1α expression (Lira, Benton, Yan, & Bonen, 2010). ROS are involved in p38 MAPK and AMPK activation and the consequent regulation of PGC‐1α expression. In addition, increased NAD during exercise activates SIRT1, which consequently activates PGC‐1α (Kang, O'Moore, Dickman, & Ji, 2009).

The endogenous protective molecules shown in Table 1 can also regulate PGC‐1α. FGF21, a hormone‐like member of the FGF family, is induced by endoplasmic reticulum (ER) stress, mitochondrial dysfunction, and autophagy (Suwa et al., 2003). FGF21 controls energy metabolism by enhancing energy expenditure, ameliorates age‐related metabolic disorders such as atherosclerosis, obesity, and T2DM (Suwa et al., 2003), upregulates hepatic PGC‐1α expression (Potthoff et al., 2009), increases NAD levels leading to the activation of SIRT1 and the deacetylation of PGC‐1α that consequently activates PGC‐1α in adipocytes (Chau, Gao, Yang, Wu, & Gromada, 2010), and enhances SIRT1 binding to liver kinase B (LKB1), which decreases LKB1 acetylation and subsequently induces the activation of AMPK in cardiomyocytes (Wang, Wang, Zhang, Liu, & Gu, 2017). The transgenic overexpression of FGF21 extends the lifespan of mice by blunting the growth hormone/insulin‐like growth factor 1 signaling pathway in the liver (Zhang et al., 2012). Among the isoforms of pyruvate kinase, PKM2 has been extensively studied due to its important role in cancer metabolism (Alves‐Filho & Palsson‐McDermott, 2016). A recent study demonstrated that the podocyte‐specific deletion of PKM2 accelerated albuminuria in streptozotocin‐induced diabetic mice and that TEPP‐46, a small molecule PKM2 activator, reversed hyperglycemia‐induced elevation in toxic glucose metabolites and mitochondrial dysfunction by increasing the PGC‐1α level (Qi et al., 2017). In addition, VEGF, one of the most important endogenous proangiogenic and prosurvival factors, can respond to hypoxia under normal physiological conditions (Schrijvers et al., 2004). PGC‐1α induces VEGF by coactivating the transcription factor ERR on an enhancer located in the first intron of the VEGF gene in myotubes (Arany et al., 2008). VEGF‐A‐knockout mice show losses of podocyte foot processes and endothelial cell fenestrations, suggesting a crucial role for VEGF in maintaining the function of the glomerular filtration barrier (Sison et al., 2010). In contrast, VEGF expression correlates with fibrotic markers in diabetic kidneys (Kinashi et al., 2017), and a selective VEGFR‐3 inhibitor ameliorates diabetic kidney injury in *db/db* mice (Hwang et al., 2019). Thus, the protective effect of VEGF in the kidney is controversial.

Peroxisomes play an important role in FAO. PGC‐1α has been suggested to play a critical role in the regulation of peroxisomal function and biogenesis (Bagattin, Hugendubler, & Mueller, 2010; Huang et al., 2017). The ectopic expression of PGC‐1α increases the levels of peroxisomal β‐oxidation‐related genes (including acyl‐CoA oxidase‐1 and enoyl‐CoA hydratase/3‐hydroxyacyl‐CoA dehydrogenase) and genes involved in peroxisomal biogenesis (such as Pex11α, Pex11β, Pex13, and Pex16). The detailed mechanism of PGC‐1α‐induced peroxisomal biogenesis remains unclear.

PGC‐1α may act as an endogenous regulator in autophagy. PGC‐1α induces not only mitochondrial biogenesis but also autophagy/mitophagy in muscle following acute exercise. Autophagy‐related genes such as microtubule‐associated protein 1 light chain 3 and sequestosome 1 are induced by exercise but attenuated in the skeletal muscle of PGC‐1α‐knockout mice (Vainshtein, Tryon, Pauly, & Hood, 2015).

While PGC‐1α increases ROS in different organelles, including the mitochondria, peroxisomes, and ER, it also stimulates the ROS scavenging pathway to balance ROS production and detoxification. Manganese superoxide dismutase (MnSOD) and glutathione peroxidase‐1, major mitochondrial components involved in ROS metabolism, are induced by at least fivefold in C2C12 myotubes expressing PGC‐1α. PGC‐1α also increases the expression of uncoupling protein 2 (UCP2) and UCP3, which protect mitochondria against ROS stress (St‐Pierre et al., 2003), leading to a redox balance in response to oxidative stress.

## 5 PROTECTIVE EFFECT OF PGC‐1α IN KIDNEY DISEASE

We first summarize the data demonstrating the protective role of PGC‐1α in acute kidney injury (AKI) followed by chronic kidney injury (Table 2).

**Table 2:** Protective effects of PGC‐1α on kidney injury

Disease models	Altered metabolic change	References
Cisplatin‐induced AKI Folic acid‐induced AKI Ischemia/reperfusion‐induced AKI LPS‐induced AKI	Autophagy Fatty acid oxidation Mitochondrial biogenesis	Lempiainen et al. (2013), Portilla et al. (2002), Ruiz‐Andres et al. (2016), Smith et al. (2015), Tran et al. (2011), Tran et al. (2016)
*db/db* diabetic mice Kidney fibrosis	Fatty acid oxidation Mitochondrial biogenesis Mitochondrial oxidative stress	Han et al. (2017), Hong et al. (2014), Kang et al. (2015), Kim, Lee, et al., (2013), Kim, Lim, et al. (2013), Long et al. (2016), Yuan et al. (2018), Zhang et al. (2018)
Aged mice	Fatty acid oxidation Mitochondrial biogenesis	Chung et al. (2018), Svensson et al. (2016)

### 5.1 PGC‐1α in AKI

The elderly are vulnerable to AKI, showing high mortality due to their decreased ability to adapt and regenerate. AKI is a crucial risk factor in kidney disease progression (Jung, Choi, Song, & Ahn, 2018; Oh, 2016). The mechanism underlying the elderly's increased susceptibility to CKD after AKI is unclear.

AKI caused by diverse etiologies is characterized by mitochondrial dysfunction. Accordingly, the activation of PGC‐1α is beneficial in AKI (Lynch et al., 2018). The expression level of PGC‐1α is decreased in various models of AKI, including cisplatin‐ (Morigi et al., 2015; Portilla et al., 2002), folate‐ (Ruiz‐Andres et al., 2016), ischemia/reperfusion (IR)‐ (Lempiainen et al., 2013; Tran et al., 2016), and lipopolysaccharide (LPS)‐induced kidney injuries (Smith, Stallons, Collier, Chavin, & Schnellmann, 2015; Tran et al., 2011). PGC‐1α‐knockout mice exhibit worse kidney function, greater fat accumulation, and more tubular injury following IR injury than wild‐type (WT) mice (Tran et al., 2016). Tubule‐specific PGC‐1α‐knockout mice exhibit normal basal kidney function but more persistent injury after LPS treatment (Tran et al., 2011). Tubule‐specific PGC‐1α‐overexpressing mice show increased levels of NAD, nicotinamide (NAM), and NAD synthetic enzymes, with higher resistance to ischemia (Tran et al., 2016). The reactivation of PGC‐1α using transgenic overexpression enhances recovery from kidney injury in various animal models of AKI (Lynch et al., 2018). These results suggest that the restoration of PGC‐1α in the kidney may be essential for functional recovery from AKI. Interestingly, CR ameliorates IR‐induced AKI (Lempiainen et al., 2013).

Increased levels of inflammatory mediators in AKI downregulate PGC‐1α through histone deacetylation. Upon TWEAK stimulation, NF‐κB Rel A directly binds to the promoter of the PGC‐1α gene (Figure 4) to recruit histone deacetylase corepressor proteins, leading to histone deacetylation and chromatin packing that suppress PGC‐1α expression (Ruiz‐Andres et al., 2016). Systemic LPS exposure leads to the activation of TLR4, which in turn initiates signaling through the TPL2/MAPK/ERK pathway, leading to a decrease in PGC‐1α mRNA (Smith et al., 2015).

### 5.2 PGC‐1α in CKD

**Figure 3:**
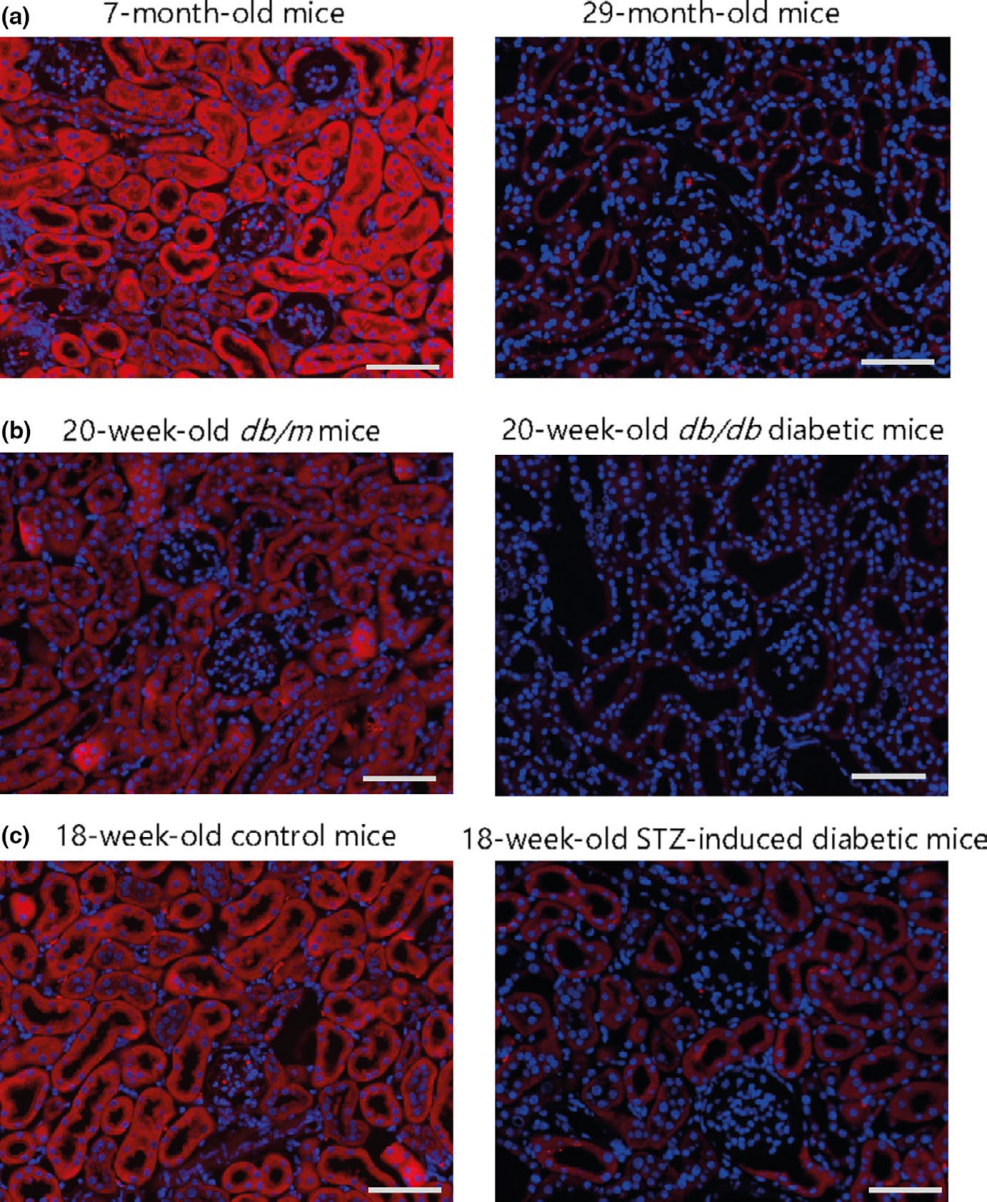
PGC‐1α expression in the kidneys of aged or diabetic C57BL/6J mice. (a) Kidney sections from 7‐month‐old and 27‐month‐old mice were examined. (b) Kidney sections from 20‐week‐old db/m or db/db mice were examined. (c) Diabetes was induced by the intraperitoneal injection of 50 mg/kg STZ for 5 days, and kidney sections were examined. (a–c) Paraffin‐embedded kidney sections were subjected to immunofluorescence staining using an anti‐PGC‐1α antibody (1:100; ab54481; Abcam) and anti‐rabbit Alexa Fluor 588 (1:1,000; A11036; Invitrogen). Nuclei were stained with DAPI (Hwang et al., 2012). Images were taken using a Zeiss ApoTome Axiovert 200 M microscope (Carl Zeiss Microscopy GmbH). Scale bar indicates 50 μm. Representative images are shown

Kidney specimens from CKD patients show decreased PGC‐1α expression (Han et al., 2017; Sharma et al., 2013), which is associated with a decreased GFR in human kidney fibrosis (Lemos et al., 2018). PGC‐1α is decreased in various models of CKD, including unilateral ureteral obstruction‐induced fibrosis (Han et al., 2017), *db/db* diabetic mice (Hong et al., 2014; Kim, Lee, et al., 2013; Kim, Lim, et al. 2013; Long et al., 2016; Yuan et al., 2018; Zhang, Liu, Zhou, Wang, & Chen, 2018), and streptozotocin‐induced diabetic mice (Kwon et al., 2017). Our results also show that PGC‐1α expression is significantly decreased in diabetic mice (Figure 3). The tubule‐specific overexpression of PGC‐1α ameliorates Notch‐induced kidney injuries, such as apoptosis, impaired mitochondrial morphology and the FAO pathway, and fibrosis (Han et al., 2017). Thus, restoring PGC‐1α activity could be a promising treatment strategy against CKD.

**Figure 4:**
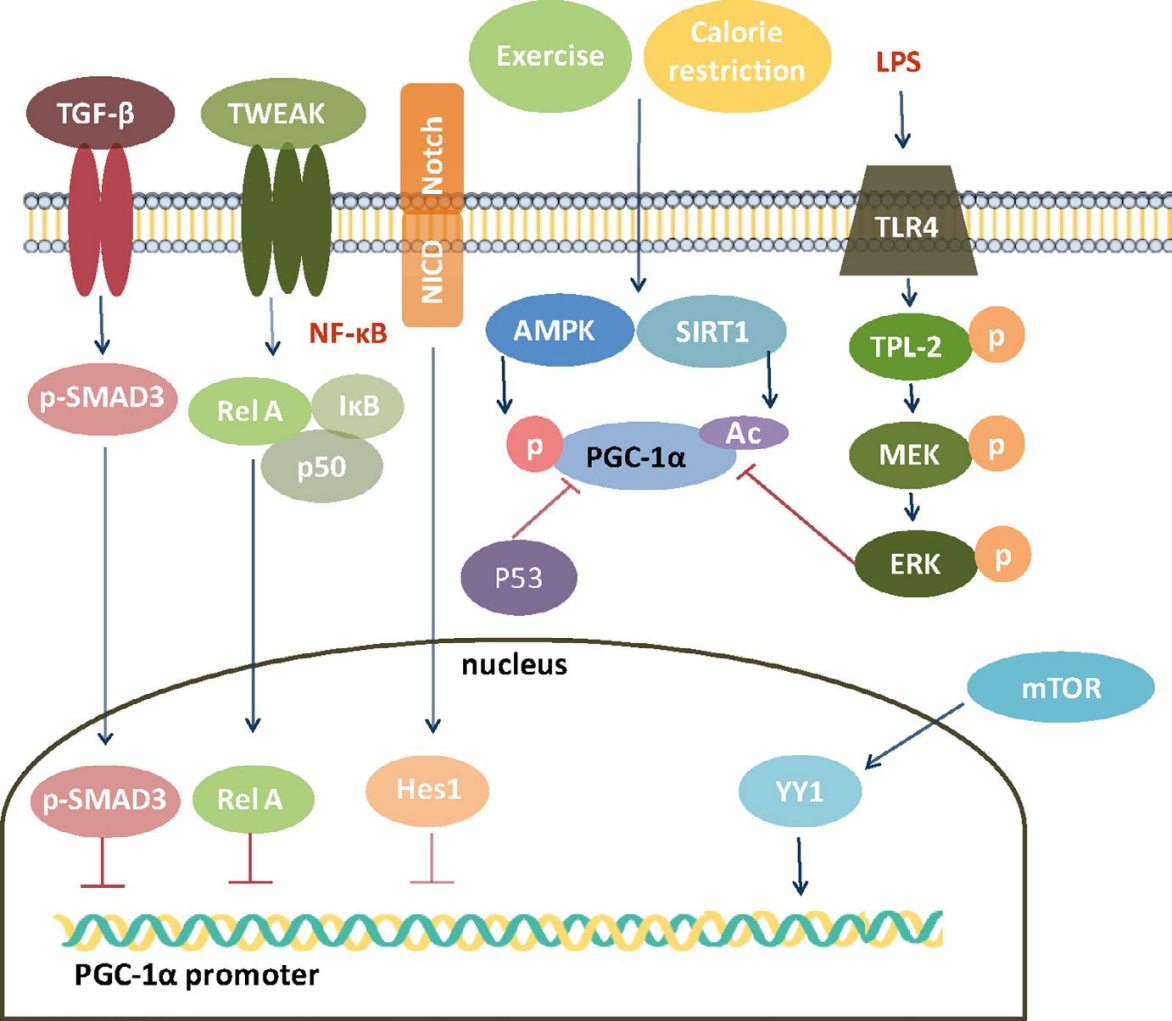
Regulation of PGC‐1α. TGF‐β, tumor necrosis factor‐like weak inducer of apoptosis (TWEAK), and Notch can repress PGC‐1α promoter activity by the SMAD3, Rel A, NF‐kB, and Hes1 pathways, respectively. On the other hand, exercise and calorie restriction activate PGC‐1α, while the activation of P53 and ERK can suppress PGC‐1α activation. mTOR‐induced YY1 increases PGC‐1α promoter activity. ERK, extracellular signal‐regulated kinase; NICD, Notch intracellular domain; MEK, mitogen‐activated protein kinase; TPL‐2, tumor progression locus 2; YY1, yin‐yang 1

As shown in Figure 4, TLR4 and NF‐*κ*B mediate diabetes‐induced PGC‐1α downregulation (Yuan et al., 2018). Transcriptional repressor Hes1 (a downstream target of fibrotic Notch signaling) directly binds to the PGC‐1α promoter region (Han et al., 2017). TGF‐β inhibits the transcription of PGC‐1α in a SMAD3‐dependent manner, leading to FAO in kidney fibrosis (Kang et al., 2015). However, taurine‐upregulated gene 1 (Tug1), an evolutionarily conserved long intergenic noncoding RNA, binds directly to an R/S‐rich region in the C‐terminal domain of PGC‐1α and enhances its expression (Long et al., 2016). The podocyte‐specific overexpression of Tug1 ameliorates mitochondrial dysfunction in *db/db* diabetic kidney (Long et al., 2016). High‐glucose‐induced PGC‐1α downregulation and ROS accumulation in mesangial cells can be reversed by PGC‐1α overexpression (Zhang et al., 2018). These findings suggest that PGC‐1α may have beneficial effects in glomeruli, although PGC‐1α is the most abundant in kidney tubules.

## 6 ROLE OF PGC‐1α IN AGING

**Figure 5:**
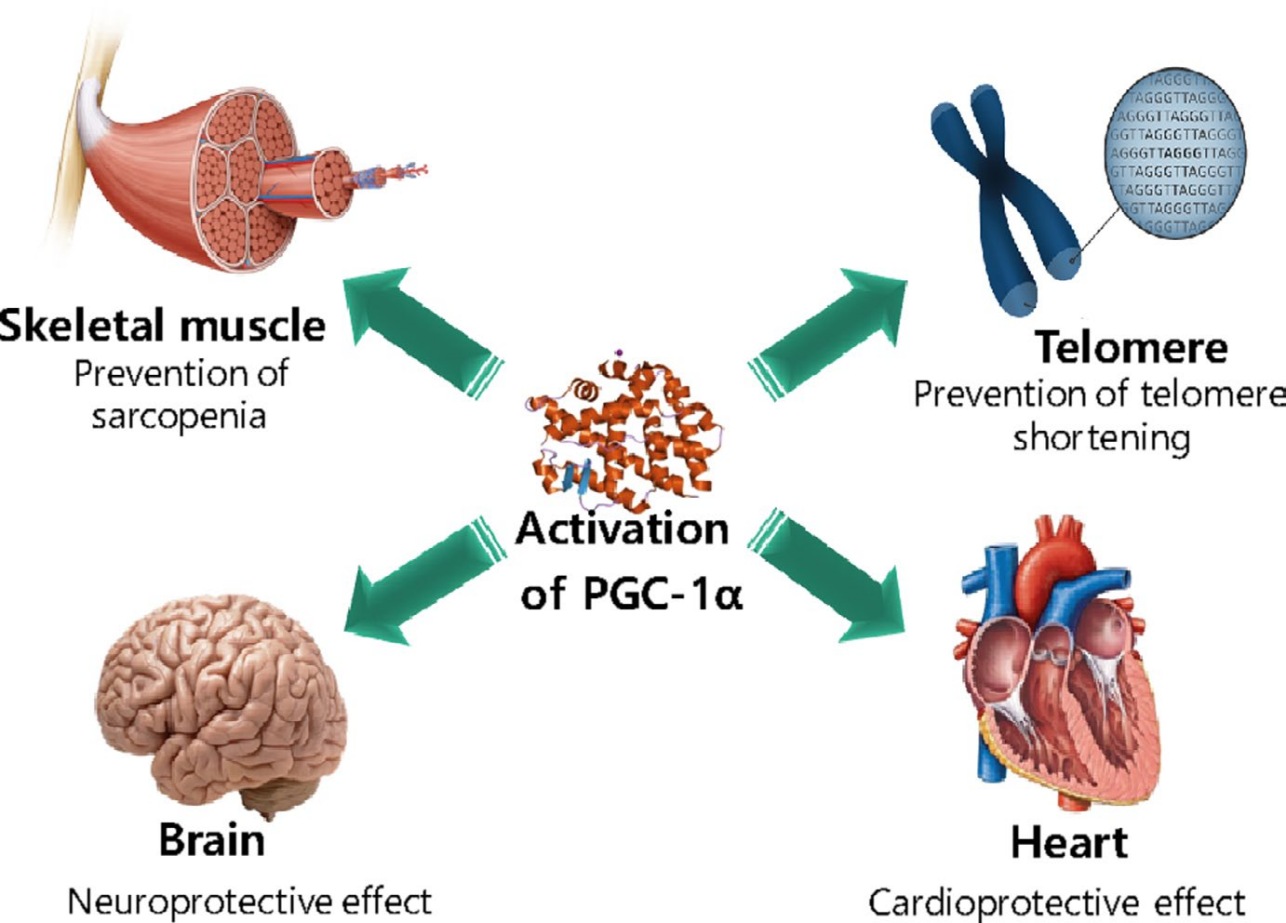
Antiaging effect of PGC‐1α activation on the muscle, heart, brain, and telomeres

Increasing evidence implicates the association of PGC‐1α and antiaging in various organs (Figure 5).

### 6.1 PGC‐1α in telomere shortening

Telomeres are gradually shortened by continued cell division. They finally enter a dysfunctional state, leading to cellular growth arrest and senescence. Telomerase reverse transcriptase (TERT) deficiency in mice results in the dysfunction and shortening of telomeres with DNA damage (Sahin et al., 2011). PGC‐1α expression is decreased in TERT‐knockout mice (Kang et al., 2018; Sahin et al., 2011). PGC‐1α deletion induces telomere malfunction, DNA damage, cellular senescence, and increased p53 levels**.** Conversely, the ectopic expression of PGC‐1α coactivates TERT transcription and reverses telomere malfunction and DNA damage (Xiong, Patrushev, Forouzandeh, Hilenski, & Alexander, 2015).

Mechanistically, PGC‐1α directly increases TERT expression (Xiong et al., 2015). There are multiple conserved PGC‐1α‐coactivated DNA‐binding elements of transcriptional factors within the rat, mouse, and human TERT promoter regions. Alpha lipoic acid, a nondispensable mitochondrial cofactor, upregulates PGC‐1α‐dependent TERT and Nrf‐2‐mediated antioxidant/electrophile‐responsive element signaling cascades, counteracting high‐fat diet (HFD)‐induced age‐dependent arteriopathy.

Telomere dysfunction can activate p53, which in turn binds to and represses PGC‐1α promoters, leading to mitochondrial impairment in the liver and heart in age‐related dilated cardiomyopathy, defects in hepatic gluconeogenesis, and reduced hematopoietic stem cell capacity to reconstitute (Sahin et al., 2011).

### 6.2 PGC‐1α in the heart, muscle, and brain

The heart requires a constant flux of ATP to maintain contractile function. Increasing evidence has shown that energetic defects contribute to the development of heart failure (Ren, Pulakat, Whaley‐Connell, & Sowers, 2010; Rowe, Jiang, & Arany, 2010). The mitochondrial mass comprises one‐third of the adult heart. PGC‐1α has recently emerged as a powerful regulator of mitochondrial biology in the heart (Rowe et al., 2010). Mitochondrial dysfunction is observed in human cardiomyopathy and most animal models of heart failure (Ide et al., 2001; Rowe et al., 2010; Weiss, Gerstenblith, & Bottomley, 2005). PGC‐1α is decreased in various in vivo models of heart failure, including transverse aortic constriction (TAC) in mice (Arany et al., 2006; Huss et al., 2007) and congestive heart failure in rats (Garnier et al., 2003), suggesting that decreased PGC‐1α is a common feature of cardiac diseases. Aging is involved in cardiac dysfunction in mice. PGC‐1α knockout accelerates cardiac failure through hemodynamic challenge in TAC in mice (Arany et al., 2006). Isolated working hearts from PGC‐1α‐knockout mice show decreased FAO and reduced cardiac power (Lehman et al., 2008). Mechanistically, ERRα‐knockout mice show a very similar phenotype to PGC‐1α‐knockout mice, with chamber dilatation and reduced left ventricular fractional shortening after TAC (Huss et al., 2007), underscoring the central role of ERRα in PGC‐1α biology. Decreased PGC‐1α expression has also been observed in the hearts of the elderly. Although the aging process is not exacerbated, young mice with PGC‐1α knockdown partially mimic age‐related impairments in mitochondrial gene expression. On the other hand, the moderate overexpression of PGC‐1α prevents numerous age‐related remodeling changes in the heart as well as the expression of various genes involved in mitochondrial biogenesis, dynamics, metabolism, calcium handling, and contractility (Whitehead, Gill, Brink, & Handschin, 2018). Since heart‐specific PGC‐1α overexpression exhibits dilated myopathy in mice (Lehman et al., 2000), this plausible adverse effect in the heart should be considered when developing therapeutic agents targeting PGC‐1α.

Failing vasculature is another major factor leading to the development of cardiovascular diseases during aging. PGC‐1α deletion accelerates vascular senescence (Wenz, 2011). PGC‐1α expression is decreased in human atherosclerosis (McCarthy et al., 2013). PGC‐1α‐deficient mice develop vascular senescence, which mainly occurs in vascular smooth muscle cells (Kroller‐Schon et al., 2013; Xiong, Salazar, Patrushev, & Alexander, 2011). Endothelial dysfunction is an early feature of chronic cardiovascular diseases (Meigs, Hu, Rifai, & Manson, 2004). The overexpression of PGC‐1α in endothelial cells reduces ROS levels and rescues ROS‐mediated mitochondrial toxicity and cellular apoptosis (Valle, Alvarez‐Barrientos, Arza, Lamas, & Monsalve, 2005; Won et al., 2010). The overexpression of PGC‐1α in endothelial cells induces the expression of MnSOD. PGC‐1α transcriptional activity at the MnSOD promoter requires a functional FOXO site (Olmos et al., 2009).

A loss of muscle mass, known as sarcopenia, is another serious health problem in the elderly. Skeletal muscle with aging exhibits mitochondrial electron transport chain defects, the accumulation of oxidative stress markers, and mutations in somatic mitochondrial DNA (mtDNA). The causative role of decreased PGC‐1α expression in age‐associated insulin resistance in muscle has been established. First, the expression of PGC‐1α along with COX activity, an index of mitochondrial content, in the soleus muscle gradually decreases from 6 to 24 months in rats (Sczelecki et al., 2014). Second, an integrated analysis of omics data from muscle‐specific PGC‐1α‐knockout mice and WT controls aged up to 2 years demonstrated that approximately 35% of genes regulated by PGC‐1α may play a role in the molecular pathway of muscle aging (Sczelecki et al., 2014). The same study suggested that the loss of PGC‐1α may be insufficient to accelerate insulin resistance but may promote glucose intolerance combined with advanced age. Third, the overexpression of PGC‐1α in skeletal muscle results in the molecular features of muscle similar to those from young mice, with mild but significant effects on the median lifespan in female mice and the maximal lifespan in male mice, suggesting muscle remodeling in younger muscle (Garcia et al., 2018). Finally, exercise upregulates PGC‐1α transcription and activity (Kupr & Handschin, 2015) through various pathways, including cytosolic Ca^+^. Thus, the beneficial effect of exercise may depend on PGC‐1α. Skeletal muscle‐specific PGC‐1α overexpression in mice has shown controversial results. It beneficially induces the conversion of type IIb muscle fiber to type I muscle fiber, rendering resistance to fatigue through oxidative metabolism (Lin, Wu, et al., 2002). On the other hand, it does not exhibit a protective effect in the HFD‐induced insulin‐resistant mouse model (Choi et al., 2008).

Alzheimer's disease (AD), Parkinson's disease (PD), and Huntington's disease are age‐dependent degenerative diseases of the central nervous system. PGC‐1α expression is decreased in patients with PD (Su et al., 2015; Zheng et al., 2010) and AD (Qin et al., 2009). PGC‐1α is decreased in various in vivo models of neurodegenerative diseases, including Tg2576 mice (an experimental AD model, Qin et al., 2009) and mutant human α‐synuclein transgenic mice (an experimental PD model, Su et al., 2015). PGC‐1α‐knockout mice mirror some aspects of premature brain aging, including ultrastructural alterations in the ER and mitochondria (Ciron et al., 2015). PGC‐1α overexpression restores mitochondrial morphology, oxidative stress detoxification, and basal respiration, consistent with the observed neuroprotection against α‐synuclein toxicity (Ciron et al., 2015). The reconstitution of exogenous PGC‐1α expression attenuates hyperglycemia‐induced amyloidogenic Aβ peptide accumulation (Qin et al., 2009). These data suggest that PGC‐1α may be a therapeutic target against neurodegenerative diseases, including brain aging. Mechanistically, decreased PGC‐1α expression might promote Aβ amyloidogenesis through FoxO3α‐mediated responses in AD (Qin et al., 2009; Tsunemi & La Spada, 2012). Methylation levels are negatively correlated with PGC‐1α mRNA levels (Barres et al., 2009; Teyssier, Ma, Emter, Kralli, & Stallcup, 2005). A previous analysis of human brain samples indicated that PD is associated with increased methylation of the PGC‐1α promoter and the reduced expression of PGC‐1α. The unfolded protein response mediates the recruitment of DNA methyltransferases such as DNMT3A to the nucleus to catalyze the methylation of the PGC‐1α promoter (Su et al., 2015).

PGC‐1α may play a critical role in the regulation of peroxisomal biogenesis (Bagattin et al., 2010) through an unknown mechanism, as stated above. Peroxisomal biogenesis is involved in the aging process in yeast and *C. elegans* (Cipolla & Lodhi, 2017; Lefevre, Kumar, & van der Klei, 2015; Weir et al., 2017). However, further studies with mammalian models are warranted to investigate the interactions between PGC‐1α and peroxisomal biogenesis in aging.

### 6.3 PGC‐1α in kidney aging

PGC‐1α expression is also decreased in the kidneys of aged mice (Lim et al., 2012). Our results show that PGC‐1α expression is decreased in 29‐month‐old mice compared to 7‐month‐old mice (Figure 3). The precise role of PGC‐1α against kidney aging is unclear.

Fenofibrate, a PPARα agonist, activates SIRT1 and AMPK, resulting in the increased expression of PGC‐1α and ERRα and the amelioration of mitochondrial dysfunction in aged kidneys. Fenofibrate improves kidney function, proteinuria, glomerulosclerosis, tubular interstitial fibrosis, inflammation, and apoptosis in age‐related kidney injury (Kim et al., 2016).

Given that paired‐box gene 8 (PAX8) is localized in epithelial cells in all segments of kidney tubules in adult kidneys (Tong et al., 2009), nephron‐specific inducible PGC‐1α‐knockout (NiPKO) mice can be generated by crossing transgenic Pax8rtTA‐(tetO‐cre)‐LC1 mice with mice harboring floxed PGC‐1α alleles (PGC‐1α fl/fl). NiPKO mice exhibit a mild loss of sodium in urine, which is exacerbated in aged and HFD‐fed mice, suggesting the beneficial role of tubular PGC‐1α in sodium homeostasis under basal conditions, aging, and metabolic stress. In addition, NiPKO mice develop exacerbated kidney steatosis on a HFD (Svensson, Schnyder, Cardel, & Handschin, 2016).

## 7 EFFECT OF ANTIAGING DRUGS ON PGC‐1α ACTIVATION

ZLN005, a small molecule discovered by luciferase assays, upregulates PGC‐1α transcription (Zhang, Shao, et al., 2013; Zhang, Zhou, et al., 2013). ZLN005 increases the mRNA expression of PGC‐1α in skeletal muscle myotubes. ZLN005 regulates PGC‐1α transcription through muscle cell‐specific transcription factors such as MEF2 and improves insulin resistance and dyslipidemia in *db/db* diabetic mice (Zhang, Shao, et al., 2013; Zhang, Zhou, et al., 2013). Additionally, ZLN005 exhibits neuroprotective and retinoprotective effects in mice (Satish, Philipose, Rosales, & Saint‐Geniez, 2018; Xu et al., 2018). However, the cardioprotective effect of ZLN005 is dependent on SIRT1 but not PGC‐1α activation (Li et al., 2016). Further research is needed to elucidate the pharmacologic target of ZLN005.

CR modulates metabolic pathways, leading to the activation of PGC‐1α (Figure 2b), SIRT1, and AMPK and the inhibition of mammalian target of rapamycin (mTOR) (Martin‐Montalvo et al., 2013). Thus, agents that modulate not only PGC‐1α but also AMPK, SIRT1, and the mTOR pathway can be considered CR mimetics (Handschin, 2016). In this respect, resveratrol (Baur et al., 2006), SRT1720 (Minor et al., 2011), metformin (Martin‐Montalvo et al., 2013), rapamycin (Anisimov et al., 2010; Fischer et al., 2015; Harrison et al., 2009; Hurez et al., 2015; Miller et al., 2014; Ramos et al., 2012; Wu et al., 2013), the NAD precursor (Fang et al., 2016; Zhang et al., 2016), and d‐glucosamine (Weimer et al., 2014) have been suggested as CR mimetics that can extend the lifespan in mice, as summarized in Table 3.

**Table 3:** Effects of antiaging drugs on PGC‐1α activation

Medication	Lifespan	PGC−1α activation	Organs	References
D‐Glucosamine	Increased	Not shown	Liver	Weimer et al. (2014)
Metformin	Increased	Increased (indirect)	Liver	Martin‐Montalvo et al. (2013)
NAD precursor	Increased	Increased	Brain, muscle	Fang et al. (2016), Zhang et al. (2016)
Rapamycin	Increased	Not shown	Heart, muscle, spleen	Anisimov et al. (2010), Fischer et al. (2015), Harrison et al. (2009), Hurez et al. (2015), Miller et al. (2014), Ramos et al. (2012), Wu et al. (2013)
Resveratrol	Increased	Increased	Liver	Baur et al. (2006)
SRT1720	Increased	Increased	Liver	Minor et al. (2011)

### 7.1 Resveratrol

Resveratrol is a naturally occurring polyphenol with anti‐inflammatory, antioxidative, antidiabetic, and neuroprotective effects (Pezzuto, 2018). The resveratrol‐activated SIRT1 pathway is associated with deacetylating activity, thereby resulting in alterations in various downstream regulators, such as PGC‐1α (Pannu & Bhatnagar, 2019). Increased SIRT1 activity triggered by elevated NAD levels increases the transcriptional activity of PGC‐1α (Rodgers et al., 2005). However, resveratrol does not bind to the native peptide of SIRT1 or full‐length protein substrates. Irrespective of the direct target of resveratrol, SIRT1 remains one of the most extensively studied targets associated with the antiaging effect of resveratrol (Pezzuto, 2018).

### 7.2 SRT1720

SRT1720, an analog of resveratrol, is an allosteric activator of SIRT1. SRT1720 has a low *K*_m_ and is 1,000 times more potent than resveratrol. SRT1720 promotes the deacetylation of hepatic PGC‐1α (Minor et al., 2011; Ungvari et al., 2009), exerts protective effects against UUO‐induced tubulointerstitial fibrosis (Ren et al., 2017), and blocks Klotho deficiency‐induced aging in arterial endothelial and smooth muscle cells (Gao et al., 2016).

### 7.3 Metformin

Metformin, an oral antidiabetic agent, can extend the lifespan in mice (Martin‐Montalvo et al., 2013). Hyperglycemia and hyperinsulinemia promote senescence. The antiaging effect of metformin is due to reduced insulin levels and a subsequent reduction in IGF‐1 signaling and glucose levels (Anisimov et al., 2011). Metformin can also inhibit mTOR signaling, reduce ROS, activate AMPK, and reduce DNA damage (Barzilai, Crandall, Kritchevsky, & Espeland, 2016). AMPK phosphorylates PGC‐1α, which controls glucose uptake, FAO, and mitochondrial biogenesis (Kim & Park, 2016). Metformin attenuates tubulointerstitial fibrosis and epithelial–mesenchymal transition in kidney injury (Lee et al., 2018; Thakur et al., 2015).

### 7.4 Rapamycin

Rapamycin, a macrolide immunosuppressant, acts primarily by inhibiting mTOR. Rapamycin extends the lifespan in mice (Anisimov et al., 2010; Fischer et al., 2015; Harrison et al., 2009; Hurez et al., 2015; Miller et al., 2014; Ramos et al., 2012; Wu et al., 2013). The suppression of mTOR is one of the key outputs of AMPK. Rapamycin may therefore phenocopy some effects of AMPK. Rapamycin ameliorates kidney fibrosis by blocking mTOR signaling in interstitial macrophages and myofibroblasts (Chen et al., 2012). However, mTOR can activate yin‐yang 1, a transcription factor that increases PGC‐1α promoter activity (Cunningham et al., 2007; Wang, Huang, et al., 2017).

### 7.5 Fenofibrate

Fenofibrate, a fibric acid derivative, is a drug of choice against hypertriglyceridemia and mixed dyslipidemia. It has lipid‐modifying effects through the activation of PPARα. Additionally, fenofibrate protects against age‐related changes in the kidney (Kim et al., 2016). PGC‐1α and PPARα may be involved in the kidney aging process (Chung et al., 2018).

### 7.6 NAD precursor

NAD plays a central role in energy metabolism (Bai et al., 2011). It also appears to play a role in healthy aging (Gomes et al., 2013). NAD is an important cofactor involved in physiological processes, including metabolism and DNA repair. NAD levels are decreased with age. A deterioration in NAD metabolism promotes several aging‐associated diseases (Yaku, Okabe, & Nakagawa, 2018; Yoshino, Baur, & Imai, 2018). High levels of NAD improve the lifespan in ataxia telangiectasia‐mutated (a master regulator of DNA damage)‐knockout mice (Fang et al., 2016). The salvage pathway in which NAD is synthesized from NAM is important for producing and maintaining intracellular NAD levels in mammals. NAM is imported as a dietary nutrient from various foods. It is a by‐product of NAD‐consuming enzymes such as SIRT1, poly (ADP‐ribose) polymerases, and NAD glycohydrolase (Revollo, Grimm, & Imai, 2004). It is synthesized from nicotinamide riboside (NR) (Bieganowski & Brenner, 2004), which increases the lifespan of aged C57BL/6J mice (Zhang et al., 2016). The NAD/SIRT pathway controls mitochondrial function through the deacetylation of PGC‐1α and FOXO (Chalkiadaki & Guarente, 2012). However, the regulation of PGC‐1α‐dependent NAD biosynthesis should be considered (Tran et al., 2016). PGC‐1α coordinately upregulates enzymes that synthesize NAD de novo from amino acids, whereas a PGC‐1α deficiency attenuates the de novo pathway. How PGC‐1α interacts with transcription factor(s) to induce the de novo synthesis of NAD is unclear (Tran et al., 2016). Exogenous NAM improves kidney NAD levels, fat accumulation, and function in postischemic PGC‐1α‐knockout mice (Tran et al., 2016).

### 7.7 Other candidates

2‐Deoxy‐d‐glucose inhibits glycolysis and reduces the ingestion, uptake, and metabolism of lipids and carbohydrates, resembling CR (Ingram & Roth, 2015). 2‐Deoxy‐d‐glucose extends the lifespan of *C. elegans* (Schulz et al., 2007). However, it has toxic effects on cardiac tissue (Minor et al., 2010). d‐glucosamine, an inhibitor of glycolysis, can also extend the lifespan of mice by enhancing mitochondrial biogenesis (Weimer et al., 2014).

## 8 LIMITATIONS OF PGC‐1α OVEREXPRESSION

PGC‐1α is strongly induced in the livers of fasting mice and mice with insulin action deficiency, such as streptozotocin‐induced diabetes, *ob/ob*, and liver IR‐knockout mice. The overexpression of PGC‐1α in both in vitro hepatocyte cultures and in vivo Wistar rats strongly enhances gluconeogenic enzymes, including phosphoenolpyruvate carboxykinase (PEPCK) and glucose‐6‐phosphatase, leading to increased glucose levels (Yoon et al., 2001). As summarized in Section 6.2, heart‐ and muscle‐specific PGC‐1α overexpression can result in dilated myopathy (Lehman et al., 2000) and the lack of a protective effect in HFD‐induced insulin resistance (Choi et al., 2008), respectively. However, utilizing the proper endogenous protective molecules summarized in Table 1 may overcome the limitations of PGC‐1α overexpression.

## 9 PROSPECTS AND FUTURE DIRECTIONS

We summarized recent studies providing evidence for PGC‐1α as a potential therapeutic target against not only AKI and CKD but also kidney aging. A decrease in PGC‐1α expression is observed in animal models of kidney diseases as well as samples from humans with kidney diseases. Accompanying metabolic dysregulation has been commonly observed in aged and diseased kidneys. Conversely, the overexpression of PGC‐1α by genetic and pharmacological interventions can attenuate the progression of kidney disease. The correlation between the PGC‐1α level and the diseased state of the kidney could extend to aging since kidney aging and kidney diseases share certain key pathologic features, such as mitochondrial and peroxisomal dysfunction and dysregulated energy metabolism.

However, there are few studies on the role of PGC‐1α in kidney aging compared to those on the role of PGC‐1α in other organs, such as the heart, skeletal muscle, and brain. Further studies are thus needed to determine whether PGC‐1α could be a novel therapeutic target against kidney aging. Studies on substances that could modulate the activity and expression of PGC‐1α should also be performed for experimental and clinical applications as a therapeutic strategy.

## 10 CONCLUSION

Mitochondria and peroxisome dysfunction play important roles in CKD and share many phenotypic similarities with aging. PGC‐1α is necessary for recovery from kidney injuries and resistance against deleterious metabolic changes. Therefore, PGC‐1α might be a potential target against kidney aging. It may promote healthy aging of the kidney. This review also warrants further studies on direct PGC‐1α activators to identify potential therapeutic strategies against kidney aging. It should be noted that both the systemic elevation and hyperphysiological activation of PGC‐1α might be associated with adverse effects in the liver, heart, and muscle. Thus, therapeutic avenues targeting PGC‐1α should be specific and tightly controlled.

## CONFLICT OF INTEREST

None declared.
